# Draft Genome Sequence of *Hydrotalea flava* Strain CCUG 51397^T^

**DOI:** 10.1128/genomeA.00527-16

**Published:** 2016-06-09

**Authors:** Laura Rabelo Leite, Julliane Dutra Medeiros, Gabriel Rocha Fernandes, Flávio Araújo, Victor Satler Pylro, Anna Christina Salim, Ângela Volpini, Guilherme Oliveira, Sara Cuadros-Orellana

**Affiliations:** a René Rachou Research Center Fiocruz, Genomics and Computational Biology Group, Belo Horizonte, Minas Gerais, Brazil; b Vale Institute of Technology, Belém, Pará, Brazil

## Abstract

We report the draft genome sequence of *Hydrotalea flava* CCUG 51397^T^, the type strain of the genus *Hydrotalea* (family *Chitinophagaceae*), isolated from water samples in southern Sweden.

## GENOME ANNOUNCEMENT

*Hydrotalea flava* CCUG 51397^T^ was isolated from water samples in southern Sweden, as previously described (1). This strain is a Gram-negative, aerobic, rod-shaped, and nonflagellated cell. According to its phylogeny, based on the 16S rRNA gene sequence analysis (1), this strain showed less than 93% similarity to all described species belonging to the *Chitinophagaceae* family, which suggests the new genera *Hydrotalea* (1). Despite the strong taxonomic support for this clade, few genomes related within have been fully sequenced to date. The sequencing of the *Hydrotalea flava* genome will improve our knowledge about the genetic potential and possible adaptive traits of this taxonomic group.

Genomic DNA from *H. flava* strain CCUG 51397^T^ was obtained from the Culture Collection of the University of Göteborg (CCUG), Sweden (http://www.ccug.se). The DNA was paired-end (2 × 300-bp) sequenced on an MiSeq platform (Illumina Inc., San Diego, CA, USA) following the manufacturer’s recommendations. The quality control of the sequences was performed with Trimmomatic version 0.32 (2), yielding a total of 5,240,937 quality-filtered reads. High-quality data were *de novo* assembled with Velvet (3), using a *k*-mer size of 201, and also using SPADES (4) with a *k*-mer size of 127. To merge and improve the assembly the Mix software (5) was employed. The draft genome consists of 120 contigs, with the longest one being 288,419 bp long and having an *N*_50_ of 82,600 bp. The estimated genome size is 3.44 Mb, and the average G+C content is 39.2%.

Genome annotation was performed with the RAST server (6). We identified 3,286 protein-coding sequences and 41 predicted noncoding RNAs (39 tRNA, one 16S rRNA, and one 23S rRNA). Analysis of the gene content of *H. flava* revealed the presence of genes coding for some well-known features of the family *Chitinophagaceae*, for example, chitinase genes, rod-shape-related genes (*mreBCD*, *pbp2*, *rodA*), and gliding motility genes (*gldBCDFGHJN*). The SEED categories showed the presence of adaptive genes involved in resistance to antibiotics and toxic compounds, nitrogen metabolism (denitrification), transposable elements and stress response.

### Nucleotide sequence accession numbers.

This whole-genome shotgun project has been deposited at DDBJ/ENA/GenBank under the accession number LUHG00000000. The version described in this paper is the first version, LUHG01000000.
